# Distributional benefits of tobacco tax and smoke–free workplaces in China: A modeling study

**DOI:** 10.7189/jogh.07.020701

**Published:** 2017-12

**Authors:** Stéphane Verguet, Gillian Tarr, Cindy L Gauvreau, Sujata Mishra, Prabhat Jha, Lingrui Liu, Yue Xiao, Yingpeng Qiu, Kun Zhao

**Affiliations:** 1 Department of Global Health and Population, Harvard T.H. Chan School of Public Health, Boston, Massachusetts, USA; 2 Department of Epidemiology, University of Washington, Seattle, Washington, USA; 3 Canadian Partnership Against Cancer, Toronto, Ontario, Canada; 4 Center for Global Health Research, Saint Michael’s Hospital and University of Toronto, Toronto, Ontario, Canada; 5 China National Health Development Research Center, Beijing, China

## Abstract

**Background:**

Tobacco taxation and smoke–free workplaces reduce smoking, tobacco–related premature deaths and associated out–of–pocket health care expenditures. We examine the distributional consequences of a price increase in tobacco products through an excise tax hike, and of an implementation of smoke–free workplaces, in China.

**Methods:**

We use extended cost–effectiveness analysis (ECEA) to evaluate, across income quintiles of the male population (the large majority of Chinese smokers), the premature deaths averted, the change in tax revenues generated, and the financial risk protection procured (eg, poverty cases averted, defined as the number of individuals no longer facing tobacco–related out–of–pocket expenditures for disease treatment, that would otherwise impoverish them), that would follow a 75% increase in cigarette prices through substantial increments in excise tax fully passed onto consumers, and a nationwide total implementation of workplace smoking bans.

**Results:**

A 75% increase in cigarette prices would avert about 24 million premature deaths among the current Chinese male population, with a third among the bottom income quintile, increase additional tax revenues by US$ 46 billion annually, and prevent around 9 million poverty cases, 19% of which among the bottom income quintile. Implementation of smoking bans in workplaces would avert about 12 million premature deaths, with a fifth among the bottom income quintile, decrease tax revenues by US$ 7 billion annually, and prevent around 4 million poverty cases, 12% of which among the bottom income quintile.

**Conclusions:**

Increased excise taxes on tobacco products and workplace smoking bans can procure large health and economic benefits to the Chinese population, especially among the poor.

China, with its 300 million plus smoking population ie, nearly one–third of the world's total, can alleviate much of the global burden of tobacco–related disease by effectively implementing tobacco control policies. Since ratification of the World Health Organization’s (WHO) Framework Convention on Tobacco Control (FCTC) in 2005, China has shown poor compliance to the FCTC’s Monitor, Protect, Offer, Warn, Enforce, and Raise (MPOWER) measures [1-3]. For example, tobacco taxes remain low, contributing only 56% of final cigarette prices, and nationwide smoking bans are yet to be comprehensively implemented [3,4].

Besides significantly contributing to premature mortality, tobacco use can impose severe financial consequences for households. Out–of–pocket (OOP) health care expenditures associated with the treatment of tobacco–related disease can be impoverishing. Cigarette expenditures also form a large proportion of all household expenditures for the poor, and together with associated health care expenses arising from smoking can contribute to increased poverty rates [5-8].

China’s slow response to smoking can be attributed to a deeply engrained tobacco culture along with structural and political obstructions [3]. Nevertheless, public support for tobacco control is growing [9,10] indicating potential for scaling up policies such as tobacco tax and smoke–free workplaces. Tobacco taxation is the most effective control policy [11,12], and modeling studies that assumed taxes fully passed onto consumers found substantial health and financial gains with the lowest income groups largely benefiting [13-15]. Yet, tobacco taxation has been so far underused in China [3]. Furthermore, the country may count up to 740 million individuals exposed to secondhand smoke (SHS) [3], which causes cancers and cardiovascular diseases [16]. Though policies against SHS are formulated primarily to protect non–smokers, they can create smoke–free areas and encourage smokers to quit or smoke less. For instance, a meta–analysis showed that workplace smoking bans in four high–income countries led to an average absolute 4% reduction in smoking prevalence [17]. However, the evidence on the effectiveness of smoke–free policies in reducing smoking in general is mixed, with variable effect sizes [17-19].

In China smoke–free policies have been differentially implemented at municipal and regional levels, and prominently enforced only during major events like the Beijing Olympics [20]. Overall, the proportion of workplaces having bans could range in 2010 from 60% in Shanghai to 20% in Jiangxi [21], and would be higher than the proportion of indoor public places having bans [21]. In 2014, nationwide workplace bans were officially proposed and relayed with large media coverage. Encouragingly, the “coming into effect” of comprehensive bans in Beijing in June 2015 was hailed domestically and internationally [22]. Other Chinese cities have adopted smoke–free laws. However, many municipal regulations are not effective due to weak enforcement including partial bans allowing for example smoking in some public places [20].

Using extended cost–effectiveness analysis (ECEA) methods [14,15,23,24], the objective of this paper is to examine and compare the distributional impact of expanding two critical tobacco control policies in China: aggressive increase in the excise tax on tobacco products; and enforcement of smoking bans in workplaces. In doing so, we update a previously validated ECEA framework and analysis [14] that estimated the health benefits, change in tax revenues, and financial risk protection, by socio–economic group, in China.

## Methods

We utilize an existing ECEA analytical framework of tobacco taxation in China [14] and develop it further in simulating and comparing two key policies: (1) a large increase in excise taxes, raising the share of all applicable taxes of the retail price of tobacco products to 75%; and (2) an implementation of total smoking bans in workplaces. Taxes currently only contribute to about 56% (39% for excise taxes) of retail prices of cigarettes in China [4], which is far from the 70% excise tax contribution to the final consumer price recommended by WHO to have a large impact on cigarette consumption [1,25]. On the other hand, worksite total bans represent an essential step forward on the way to comprehensive smoke–free environments, which have been weakly enforced in China so far [20,26].

This paper builds on a former ECEA of tobacco taxation in China [14], and extends it in three important ways. First, it uses an updated set of parameters (eg, price of cigarettes, tax share as a percentage of cigarette price; Table 1). Second, it estimates smoking–related premature deaths, and cases of impoverishment and catastrophic expenditures due to OOP treatment costs of tobacco–related diseases, both critical measures of lack of financial protection commonly used by policymakers [48]. Third, it adds the examination of another key policy among the MPOWER measures [2], the enactment of smoking bans in workplaces.

**Table 1:** Inputs used in the modeling of the expansion of tobacco control policies in China.

Input	Value	Source
Male population by age group	• 0–4 y–olds: 46 223 844	[27]
	• 5–9 y–olds: 42 116 819	
	• 10–14 y–olds: 44 333 255	
	• 15–19 y–olds: 57 372 413	
	• 20–24 y–olds: 69 787 588	
	• 25–29 y–olds: 54 148 396	
	• 30–34 y–olds: 48 300 078	
	• 35–39 y–olds: 60 477 911	
	• 40–44 y–olds: 62 353 282	
	• 45–49 y–olds: 52 513 698	
	• 50–54 y–olds: 41 888 301	
	• 55–59 y–olds: 41 743 573	
	• 60–64 y–olds: 28 223 579	
	• 65–69 y–olds: 19 966 448	
	• 70–74 y–olds: 15 697 892	
	• 75–79 y–olds: 10 754 066	
	• 80–84 y–olds: 5 524 515	
	• ≥85 y–olds: 2 757 397	
Smoking prevalence per age group (% of male population)	• 15–19 y–olds: 14.0%	Authors’ calculations based on [28,29]
	• 20–24 y–olds: 48.8%	
	• 25–29 y–olds: 53.0%	
	• 30–34 y–olds: 52.2%	
	• 35–39 y–olds: 57.5%	
	• 40–44 y–olds: 68.0%	
	• 45–49 y–olds: 66.7%	
	• 50–54 y–olds: 58.0%	
	• 55–59 y–olds: 57.7%	
	• 60–64 y–olds: 47.3%	
	• 65–69 y–olds: 37.6%	
	• 70–74 y–olds: 21.0%	
	• 75–79 y–olds: 19.0%	
	• 80–84 y–olds: 17.0%	
	• ≥85 y–olds: 13.0%	
Relative smoking prevalence per income quintile	• Income quintiles I to IV: 1.14 times average per age group	[14,28]
	• Income quintile V: 0.86 times average per age group	
Cigarette consumption (cigarettes per day) per income quintile	• Income quintile I to V: 15.6, 15.5, 13.8, 12.7, 12.7	[14,28]
Price per pack (20 cigarettes) (2015 US$)	• US$ 2.00 (before excise tax increase)	[4]
	• US$ 3.50 (after excise tax increase)	
Taxes per pack (20 cigarettes) (2015 US$)	• US$ 1.12 (before: 56% of retail price)	[4]
	• US$ 2.63 (after: 75% of retail price)	
Relative smoking prevalence reduction among workers after workplace smoking ban	• 9%	[30]
Proportion of deaths among smokers attributable to smoking	• 0.50	[31]
Reduction of smoking–attributable death risk by age at quitting	• 15–19 y–olds: 96.9%	Authors’ derivations based on [31] (**Online Supplementary Document(Online Supplementary Document)**, section 1)
	• 20–24 y–olds: 94.8%	
	• 25–29 y–olds: 92.1%	
	• 30–34 y–olds: 89.2%	
	• 35–39 y–olds: 86.6%	
	• 40–44 y–olds: 83.7%	
	• 45–49 y–olds: 79.5%	
	• 50–54 y–olds: 72.9%	
	• 55–59 y–olds: 62.8%	
	• 60–64 y–olds: 49.9%	
	• 65–69 y–olds: 36.4%	
	• 70–74 y–olds: 24.7%	
	• 75–79 y–olds: 15.7%	
	• 80–84 y–olds: 9.1%	
	• ≥85 y–olds: 4.5%	
Proportion of smoking–attributable deaths per cause of death	• COPD: 11.3%	[32]
	• Stroke: 45.5%	
	• Heart disease: 22.8%	
	• Neoplasm: 20.4%	
Tobacco–related disease treatment costs (2015 US$)	• COPD: US$ 2256	[14] and based on [33-40]
	• Stroke: US$ 2197	
	• Heart disease: US$ 11 774	
	• Neoplasm: US$ 14 794	
Utilization of health care by tobacco–related disease (%)	• COPD: 33%	[14] and based on [41-43]
	• Stroke: 80%	
	• Heart disease: 81%	
	• Neoplasm: 50%	
Relative utilization of health care per income quintile	• Income quintile I to V: (0.79, 0.98, 1.00, 1.08, 1.15) times average (applies to % above)	[14] and based on [44]
Fraction of health care costs reimbursed by insurance schemes	• 48%	Authors’ derivation based on [45] (**Online Supplementary Document(Online Supplementary Document)**, section 1)
Annual income per capita (2015 US$)	• Income quintile I: 0 to US$ 992	Income distribution based on average per capita income of US$ 3039 and Gini coefficient of 0.43 [46,47]
	• Income quintile II: US$ 992 to 1870	
	• Income quintile III: US$ 1870 to 2973	
	• Income quintile IV: US$ 2973 to 4718	
	• Income quintile V: > US$ 4718	
Assumed price elasticity of demand for cigarette by age group (≥25 y–olds; 15–24 y–olds; future smokers ie, under 15 y–olds) and income quintile	• Income quintile I: –0.64; –1.28; –1.28	
	• Income quintile II: –0.51; –1.02; –1.02	
	• Income quintile III: –0.38; –0.76; –0.76	
	• Income quintile IV: –0.25; –0.50; –0.50	
	• Income quintile V: –0.12; –0.24; –0.24	

COPD, chronic obstructive pulmonary disease, y – year, d – day

We selected the policies of increases in excise taxes (i) and smoke–free workplaces (ii) as they were two essential MPOWER measures [2]. Policy (i) was chosen because China recently passed in 2015 a tobacco tax reform increasing the retail price of tobacco with tax then representing 56% of cigarette prices [4]; yet, this is far from WHO’s 70% recommendation [1,25]. Policy (ii) was chosen because of China’s recent advancements in adopting smoke–free laws. Notably, Beijing adopted in 2015 a comprehensive 100% smoke–free law for all indoor public places with high compliance rates [22,49]. More than 15 other cities since enacted similar legislations or enforced smoke–free policies, such as Shanghai and Shenzhen in 2017; nevertheless, such regulations are not always fully enforced, and China has yet to implement a nationwide smoke–free legislation. Our choice of policies highlights the potentially large benefits to be reaped as China makes nascent but important, concrete steps towards reducing its own burden and, in turn impacts the global burden of smoking.

### Study population

We examined the current Chinese male population [27] as a whole (excluding Hong Kong and Macao Special Administrative Regions). Policy impact was estimated for males only, as they disproportionately engage in smoking [28,50]: nationwide, about 53% of males smoke compared to only 2% of females at ages 15 years and above [28]. The population was structured using five–year age groups from age 0 to age 84 and a single age group for all men above age 85; and further divided into income quintiles.

National smoking prevalence of manufactured cigarettes by age group was obtained from the Global Adult Tobacco Survey (GATS) China Report 2010 for ages 15–69 [28] (**Table 1**). A study of smoking among elderly in Hong Kong was used to estimate the smoking prevalence in men above age 70 [29]. The future smoking prevalence of those under age 15 was assumed to be the prevalence among 15– to 19–year–olds; and no additional smoking initiation would take place among those above age 15. These were two conservative assumptions as the prevalence among those aged older (eg, 25– to 49–year–olds) was higher (**Table 1**).

Price elasticity of demand for cigarettes varied per income quintile. We assumed an average price elasticity of –0.38 [51], with greater elasticity among poorer smokers. We also assumed that price elasticity was twice as large in younger smokers (15– to 24–year–olds), who are more responsive to changes in prices (two–three times more) than older smokers [11,25]. Thus, we assigned 15– to 24–year–olds a 2–fold elasticity modifier across all quintiles, which we also applied to the elasticity that would affect smoking initiation for current 0–to 14–year–olds. Half of all price elasticity was apportioned to participation and half to consumption [11,25].

### Policy scenarios

The two policies, excise tax increase and workplace total bans, were independently modeled and applied uniformly nationwide.

#### Excise tax increase

Excise taxes currently only contribute to about 39% of retail prices of cigarettes in China [4]. An excise tax hike passed fully onto the consumer and resulting in a 75% increase of the cigarette pack retail price was projected, which affected smoking participation, consumption, and initiation. This led to a 75% rate of all applicable taxes (a 65% excise tax rate) on the retail price. All changes in smoking behavior except initiation were modeled as occurring in the first year of the increase. The proportion of smokers quitting and “averted” future smokers (ie, current 0– to 14–year–olds that would not initiate smoking) was calculated for each income quintile as the product of: the quintile–specific price elasticity, the proportion of the price elasticity affecting participation (one half), the youth modifier if appropriate, and the relative price increase. This proportion was further multiplied by the number of baseline smokers per age group to obtain the number of smokers who would quit or be averted.

#### Workplace total bans

The reduction in smoking following a total workplace ban was modeled as a one–time reduction in the number of baseline smokers based on a relative reduction in smoking prevalence of 9% (as used in SimSmoke China) [30]. We calculated the numbers of current smokers projected to quit and of future smokers averted by multiplying the number of baseline smokers per age group and the assumed relative reduction in prevalence.

### Policy outcomes

Impact was assessed for: averted premature deaths; additional revenues generated through excise tax hike; averted OOP expenditures due to tobacco–related disease treatment costs; prevented cases of medical impoverishment (hereafter referred as poverty cases); and prevented cases of catastrophic expenditures. Additional tax revenues were based on changes in smoking prevalence, consumption, and tax increase. Averted premature deaths among quitters were the primary health outcome. These averted deaths were then used to calculate the number of cases of poverty and catastrophic expenditures averted. All outcomes were examined by income quintile.

Tax revenues prior to policy change were calculated based on the average number of cigarettes consumed per day, the price per pack, the current tax rate (56%), and the baseline number of current smokers. After policy change, revenues were based on non–quitting smokers, reductions in consumption, and tax increases through excise tax hike (from 56% to 75% of retail price).

Smoking–related premature deaths were calculated for all male smokers currently alive. We assumed that half of all deaths among smokers were attributable to smoking [31] and that the risk of death was attenuated among former smokers based on age at quitting. Under the simplifying assumption that no current smokers would quit in the absence of policy, we calculated the number of premature deaths without policy as half (ie, 50% premature mortality rate) [31] of the baseline smoker population and of those currently under age 15 anticipated to initiate smoking. The attenuation of excess mortality risk among former smokers was modeled by age at cessation using cubic splines based on age–specific risk reductions [31]. After policy, premature deaths were calculated as 50% of continuing smokers and the age–attenuated reduction of baseline smokers quitting (Table 1). Averted premature deaths were apportioned among the four main causes of smoking–related death [32]: stroke, heart disease, neoplasms, and chronic obstructive pulmonary disease. Healthcare utilization for each cause and an adjustment per quintile were used to determine how many of those with an averted death would have incurred medical expenses. OOP expenditures were calculated by subtracting average inpatient reimbursement covered by insurance [45] from cause–specific treatment costs (see Section 1 in **Online Supplementary Document(Online Supplementary Document)**).

Per capita income [46] and Gini coefficient [47] were used to create gamma distributions for income [52,53]. Simulations generated income for each averted premature death that would have incurred medical expenses, at the quintile level. Averted cases of poverty were calculated as individuals for whom the simulated income was above US$ 1.90 per day but whose annual net income would have decreased to less than US$ 1.90 per day after paying out of pocket for disease treatment. Averted cases of catastrophic expenditures were calculated as individuals for whom OOP tobacco–related disease treatment costs would have exceeded 10% of their simulated annual income.

### Sensitivity analyses

A number of sensitivity analyses were conducted to test key scenarios and parameters (see Section 2 in **Online Supplementary Document(Online Supplementary Document)**). First, for excise tax hike, the model was run with a flat price elasticity across income quintiles (eg, –0.38). Second, we tested the impact of “brand switching” by incorporating a parameter which could capture the proportion of smokers (proportions of 0.33 and 0.75 were tested) who would respond to tobacco price increases by switching to a cheaper cigarette brand instead of quitting or decreasing consumption. This “brand switching” effect could capture for instance substitution to off–market (black market) products. Third, for workplace bans, we used an alternative effect size in assuming both an absolute reduction in smoking prevalence of 3.8% and a decrease in consumption of 3.1 cigarettes per day among continuing smokers based on a meta–analysis from four countries [17]. In this case, the absolute reduction in prevalence was further adjusted to 2.2% accounting for the proportion of worksites already having full bans (31%) [28] and restricting impact to men under age 60 that were employed (82%) [54]. Insufficient evidence however prevented from testing the differential responsiveness to worksite bans by income quintile or by age group. Yet, we tested an alternative relative prevalence reduction of 4.5% among the bottom income quintile (keeping 9% in all the other quintiles), to capture the possibility that smokers in the bottom quintile may not be employed in the formal sector where such smoking bans could be implemented. Fourth, for each policy, we selected two alternative poverty thresholds, US$ 1 and US$ 3 per day, respectively, to estimate the number of poverty cases averted.

Complete details of the mathematical derivations used and of the sensitivity analyses implemented are given in **Online Supplementary Document(Online Supplementary Document)**. All simulations used the R statistical software (http:www.r–project.org).

## RESULTS

**Figure 1:**
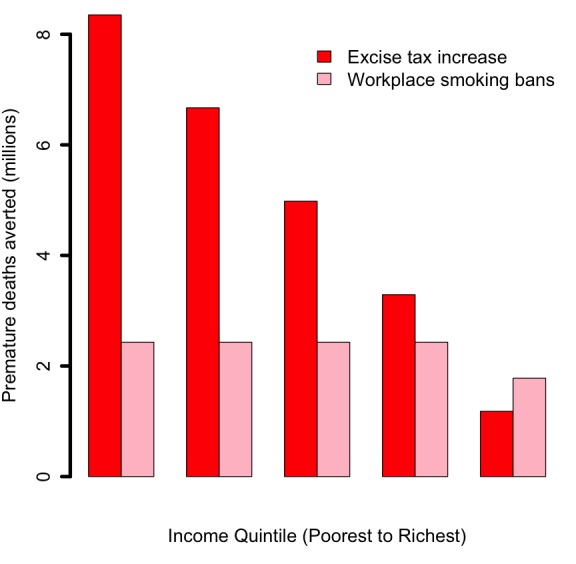
Impact of tobacco control policies (75% increase in the retail price of cigarettes through excise tax; workplace total smoking bans) in China, per income quintile, on the number of tobacco–related premature deaths averted.

**Figure 2:**
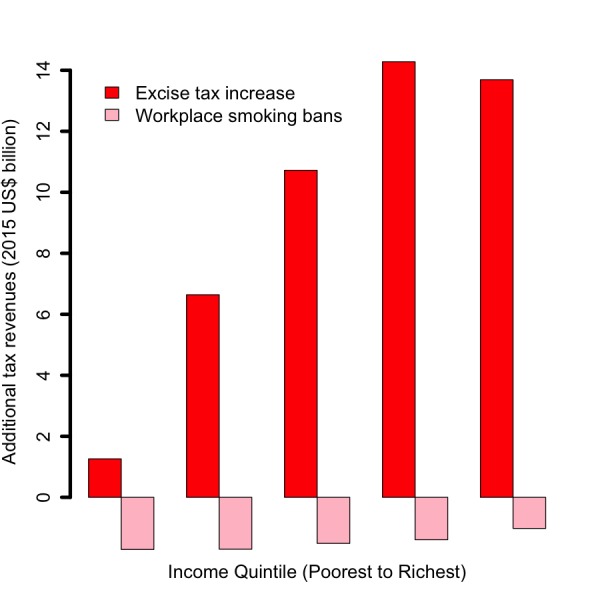
Impact of tobacco control policies (75% increase in the retail price of cigarettes through excise tax; workplace total smoking bans) in China, per income quintile, on the net change in annual tax revenues collected on cigarette sales among current smokers (15 years of age and above).

**Figure 3:**
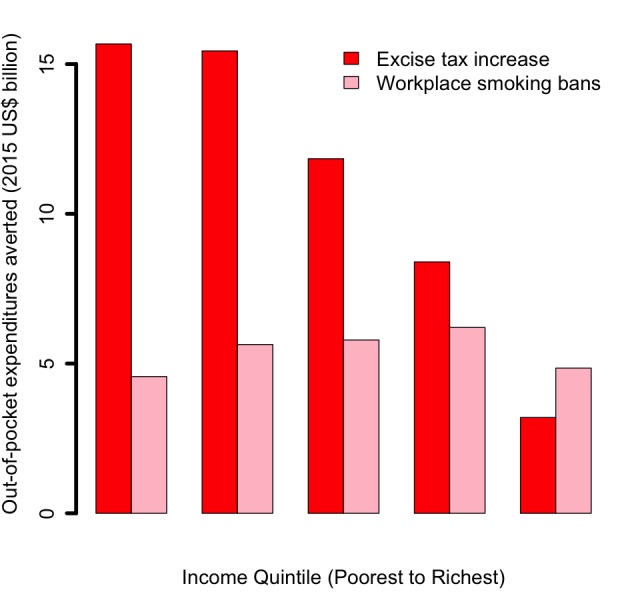
Impact of tobacco control policies (75% increase in the retail price of cigarettes through excise tax; workplace total smoking bans) in China, per income quintile, on the amount of out–of–pocket tobacco–related disease treatment costs averted.

**Figure 4:**
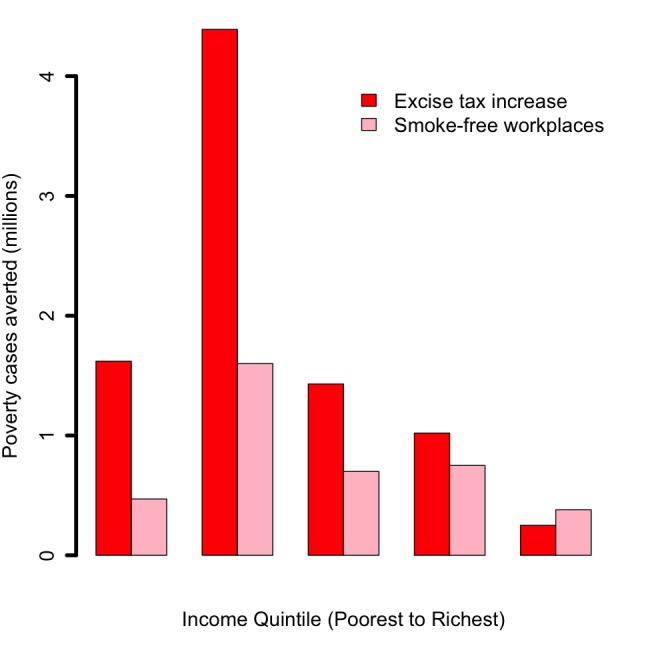
Impact of tobacco control policies (75% increase in the retail price of cigarettes through excise tax; workplace total smoking bans) in China, per income quintile, on the number of tobacco–related poverty cases averted due to the prevention of out–of–pocket tobacco–related disease treatment costs.

**Figure 5:**
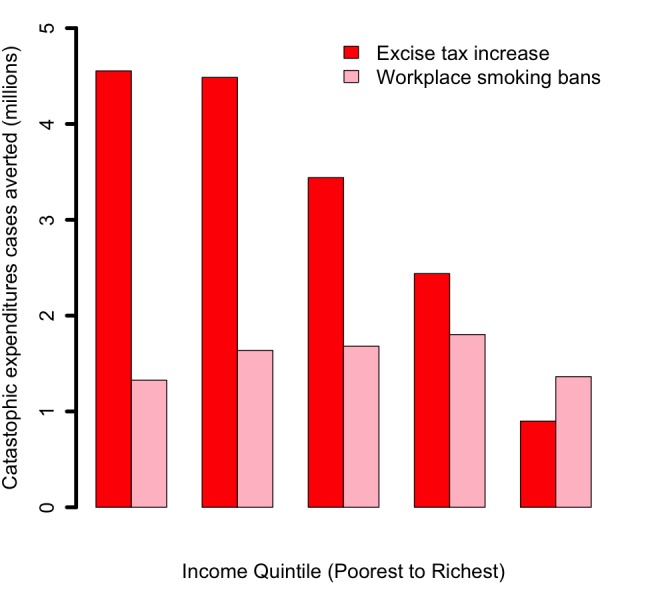
Impact of tobacco control policies (75% increase in the retail price of cigarettes through excise tax; workplace total smoking bans) in China, per income quintile, on the number of tobacco–related cases of catastrophic expenditures averted due to the prevention of out–of–pocket tobacco–related disease treatment costs.

Increasing the retail price of cigarettes by 75% (or raising the tax share as a proportion of the retail price for all applicable taxes from 56% to 75%) would reduce the number of tobacco–related premature deaths by 24 million: 61% of averted deaths would be among the bottom two income quintiles, compared to 18% among the top two quintiles (**Figure 1**). This distribution is a consequence of poorer individuals being more responsive to the significant relative change (75%) in cigarette prices, and thus quitting in larger numbers. Additional annual tax revenues raised through excise tax hike would be US$ 47 billion: 17% and 60% of these additional revenues would come from smokers from the bottom 40% and top 40% of the population income distribution, respectively (**Figure 2**). These findings are largely driven by poorer individuals being more responsive to the relative change in cigarette prices, hence quitting and smoking less cigarettes in larger numbers. The lower smoking prevalence among the top income quintile explains the slight difference in revenues between quintiles IV and V (**Figure 2**). OOP expenditures averted would be US$ 55 billion: 57% and 21% of these would be among smokers from the bottom 40% and top 40% of the income distribution, respectively (**Figure 3**). Nine million cases of poverty would be prevented, primarily among the bottom two quintiles (69%; **Figure 4**); and 16 million cases of catastrophic expenditures would be prevented, primarily among the bottom two quintiles (57%; **Figure 5**). This is a consequence of: poorer individuals being more responsive to the relative change in cigarette prices, quitting in larger numbers and facing less tobacco–related disease OOP treatment costs; a lower disposable income of poorer individuals; and the choice of the poverty threshold (see variations in the poverty cases findings when distinct poverty thresholds are used; Figure S3 in **Online Supplementary Document(Online Supplementary Document)**).

Comparatively, implementing workplace bans would yield smaller reductions in tobacco–related premature mortality and poverty cases averted (**Figures 1** and **4**). This is due to the smaller effect size (eg, among adults, 9% relative reduction in smoking prevalence for workplace bans compared with an estimated average reduction of 0.40 by 0.75 by 1/2 ~ 15% for price hike). By implementing a ban, premature deaths were estimated to decrease by 12 million and to be evenly distributed across quintiles, due to a more or less flat distribution of smoking prevalence and assumed equal responsiveness to smoking bans by income quintile. Four million cases of poverty would be prevented, primarily among the bottom two quintiles (52%), explained by the fact that poorer individuals had lower disposable income. Compared with the excise tax increase, a reduction in smoking with workplace bans would decrease tax revenues by US$ 7 billion (**Figure 2**) and will not be compensated by tax hike on tobacco products. Larger decreases (47% of the total decrease) would be observed among the bottom two income quintiles as a result of a larger number of cigarettes consumed among these groups in the first place.

**Figure 6:**
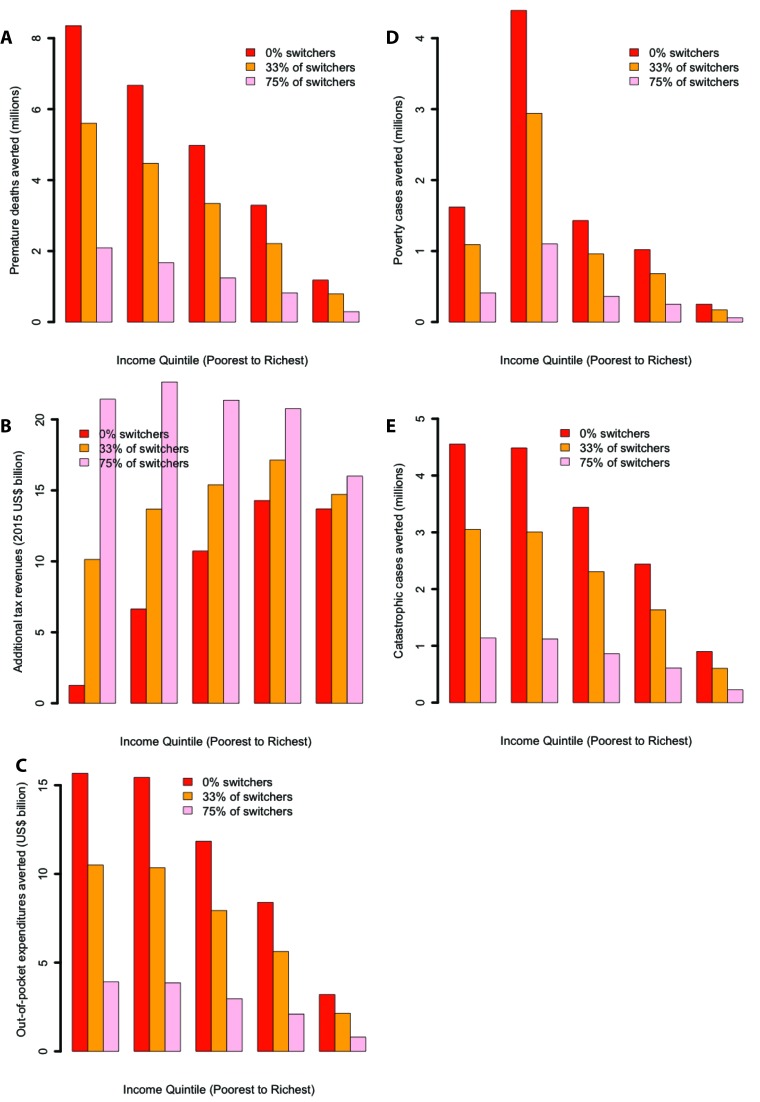
Impact of a 75% increase in the retail price of cigarettes through excise tax (proportion of smokers switching to cheaper cigarette brands, ie, “switchers”, was set at either 0%, 33%, or 75%) in China, per income quintile, on: the number of tobacco–related premature deaths averted (a); the net change in annual tax revenues collected on cigarette sales among current smokers (15 years of age and above) (b); the amount of out–of–pocket tobacco–related disease treatment costs averted (c); the number of tobacco–related poverty cases averted due to the prevention of out–of–pocket tobacco–related disease treatment costs (d); and the number of tobacco–related cases of catastrophic expenditures averted due to the prevention of out–of–pocket tobacco–related disease treatment costs (e).

Sensitivity analyses showed that policy impact was affected by a number of parameters and scenarios. First, the distributional analysis of the excise tax was largely influenced by the differential responsiveness to price changes per income quintile. Predictably, the excise tax hike progressiveness disappeared when all quintiles were given the same price elasticity (Figure S1 in **Online Supplementary Document(Online Supplementary Document)**). Assuming a flat price elasticity would equalize the number of premature deaths averted, lead to a larger share of additional taxes borne by the poor, while some pro–poor aspect of the impoverishment averted would be maintained as the poorer income quintiles would evidently still have a lower income. Second, cigarette brand switching could significantly alter the findings (**Figure 6**). Introducing brand switching produced large reductions in averted deaths (compared with the base case) equivalent to the proportion of individuals switching brands (eg, assuming 75% of smokers switch, deaths averted would decrease by 75%). Additional tax revenues through excise tax hike could increase substantially and were more evenly distributed among income quintiles, minimizing policy progressiveness. In summary, less progressiveness followed greater switching. Third, using an alternative effect size for workplace bans (absolute reduction in smoking prevalence and cigarette consumption) would alter the conclusions (Figure S2 in **Online Supplementary Document(Online Supplementary Document)**): both premature deaths and poverty cases averted would decrease; and the additional tax revenues would decrease further when smoking prevalence reduction is accompanied by consumption reduction. Likewise, a smaller prevalence reduction of 4.5% among the bottom quintile would decrease substantially (by 50%) the premature deaths averted, increase the OOP expenditures and the poverty and catastrophic cases among the poor; it would however decrease the revenue losses among the bottom income quintile (Figure S2 in **Online Supplementary Document(Online Supplementary Document)**). Fourth, we found that the poverty cases headcounts could be substantially affected when using distinct poverty thresholds (Figure S3 in **Online Supplementary Document(Online Supplementary Document)**). Expectedly, progressiveness was enhanced when the poverty threshold was reduced (eg, from US$ 3.00 to US$ 1.00 per day).

## DISCUSSION

We studied the distributional impact of expanding two tobacco control policies, aggressive increase in the excise tax on tobacco products and enforcement of workplace smoking bans, in China. On the one hand, excise tax hike passed onto the consumer in the form of a 75% retail price increase would prevent 24 million premature deaths (about 2% of China’s population) and 9 million cases of poverty, and yield an annual US$ 47 billion more in revenues. China’s poorest would experience the greatest benefits in averted deaths and impoverishments while bearing a smaller burden of the tax hike. On the other hand, instituting workplace smoking bans would have a more moderate impact on mortality and impoverishment averted. Assuming a relative decrease in smoking prevalence of 9%, 12 million premature deaths (or 1% of the Chinese population) and 4 million poverty cases could be averted, while revenues would decrease.

This analysis has shown that expanded tobacco control could promote equity at the national level in China. Yet, a subnational examination of smoking–related inequalities is required to fine tune policy. Significant geographical variations in income and health exist in China, with the West and Southwest less economically advanced, uneven access to health care between urban and rural populations, and considerable intra–urban inequality. Even in the major cities like Beijing, large health and economic gains can accrue for the poor.

Our estimates of averted premature mortality and revenue gains are consistent with previous work [13,14]. Few models on impact of workplace smoking bans exist against which to compare our results. We chose to focus on workplace bans given availability of data and also because the working population of ages 25–54 has the highest smoking prevalence and thus is of greater relevance [28]. Unfortunately, the lack of clear evidence [18] for differential responses to smoke–free policies across socio–economic groups precluded us from examining whether such policies could redress inequities observed in outcomes.

Nevertheless, our analysis presents a number of limitations. First, our estimates are sensitive to assumptions about the price elasticity of demand for tobacco. We have used an elasticity of –0.38 following norms from developed countries [11]. China has a few studies estimating price elasticity of demand for tobacco with ranges from –0.84 to –0.01 [51]. Nonetheless, we elected to use a value closer to high–income countries to get a more conservative estimate as these studies report a wide range of price elasticities. Second, our effect size for the relative reduction in prevalence due to smoking bans (eg, 9%) is limited by basing it on simulation inputs [30] that may not be generalizable. We certainly underestimated the potential impact of bans by our focus on mortality and men alone. Women could be the main beneficiaries of reduced SHS exposure [55,56], which could also lead to significant decreases in hospital admissions and associated OOP medical payments [57]. In addition, the distributional impact of smoke–free policies is unclear. In four countries, there was no correlation between socio–economic status and the introduction of smoking bans in workplaces [58]; and Dinno and Glantz [59] found that the decrease in smoking prevalence due to clean air laws in the US did not vary by socio–economic status. Third, in the past, tax increases have not generated significant behavior changes among Chinese smokers [51], which may make our model appear optimistic. For example, cigarette prices have not increased in China at the same rate as disposable incomes making them more affordable [51]. However, past excise tax increases have been low, and we emphasize here the importance of large excise tax hikes. Furthermore, cigarettes in China also have wide variation in prices, allowing consumers to switch to lower–priced brands when taxes increase [60-63]. Therefore, we have modeled different levels of brand–switching in sensitivity analyses to explore its potential impact on our estimates (**Figure 4**). This is equivalent to using a lower average price elasticity, similar to those seen in other models [13,30]. With the largest brand–switching modeled (equivalent to an average elasticity of –0.10), five million premature deaths would still be averted, but policy progressivity would be diminished. A more moderate switching parameter (corresponding to an average elasticity of –0.25) would project an averted 14 million premature deaths and preserve some progressivity. However, more research is needed for quantifying the extent of brand–switching and which smokers are more likely to switch to enact an optimum level of taxation. Fourth, since we examined the consumer perspective, one major limitation is not taking into account the role of the Chinese State Tobacco Monopoly Administration (STMA) and China’s tobacco tax structure. STMA determines cigarette prices, including the use of central government and local government taxes, and thus, tax increases do not get necessarily passed onto the retail price of tobacco products [63,64]. Excise taxes will only have an effect when increases are passed onto the consumers through higher retail prices [60,63], which is what our analysis assumed. If the tax increase were not fully (but partially) passed onto the consumers, we would still observe reductions (though diminished) in premature mortality and tobacco–related OOP spending and impoverishment. Fifth, as in all models, we had to balance interpretability and strength of existing evidence with realism. For example, we assumed that changes in smoking behaviors due to policy would occur among individuals who would otherwise not have quit on their own. Thus, we did not attempt to capture background quitting or consumption reductions. This simplifying assumption would have resulted in an overestimate of impact in presence of downward smoking trends. We also assumed that all changes in smoking behaviors were instantaneous and persisted over the life of individuals; and did not account for the fact that increased taxes may themselves be a source of impoverishment and enhance poverty, notably for those among the bottom income quintile who do no quit. Finally, for simplicity, our analysis studied one policy at a time, and thus did not model any synergies and interactions from the effects of both policies. We expect that as tobacco control in China grows and individual policies become integrated in coordinated national frameworks, evidence may be collected and research be conducted to examine complementarity of measures and their results.

Our analysis focuses on China but its findings are relevant to many other low– and middle–income countries. Other settings have already successfully implemented excise tax hikes and smoking ban policies validating our approach. Large increases in specific excise taxes can have a substantial impact on cigarette consumption [11,12,25]. For example, youth smoking is very responsive to cigarette prices as shown by data from 17 low– and middle–income countries [65]; and over 15 years, South Africa tripled cigarette prices and halved tobacco consumption with large tax hikes [66,67]. Likewise, a review [18] showed a consistent positive impact of national smoking bans on improving cardiovascular health outcomes and reducing mortality and morbidity from tobacco–related diseases, based on data from 21 countries including the middle–income countries of Argentina [68,69], Uruguay [70], Panama [71], and Turkey [72]. And a meta–analysis demonstrated that workplace bans in Australia, Canada, Germany, and the U.S led to a 4% absolute reduction in smoking prevalence [17].

Our results highlight the need to consider not only the overall impact of policies to decrease smoking but also how impact is distributed across sub–populations. More importantly, the distributional impact of tobacco control efforts provides governments with relevant evidence to make the biggest difference for the populations that most need it. We show here that increasing cigarette taxes and instituting smoke–free workplaces can potentially prevent millions from being impoverished as a consequence of smoking–related medical expenditures. Because of the structure of the existing tobacco policies and state–owned tobacco industry, we believe that a priority should be placed on full implementation of all MPOWER measures [2] including comprehensive smoking bans and large excise tax hikes. Following India’s differentially taxed cigarettes based on the length of cigarettes may help mitigate brand–switching and other compensating behaviors [73]. Higher taxes and workplace smoking bans can work well together and are complimentary: by having a mutually strengthening effect (eg, smoking bans impose social norms and enhance the price effect of taxes) they can reinforce each other to both lower consumption and bring large health and economic benefits to households. In addition, higher taxes can lead to revenue increases partially offsetting revenue losses from smoking bans. Such benefits would arise from large excise tax hikes that explicitly narrow the price differentials from top to bottom cost cigarettes, combined with total (not partial) smoking bans. In summary, the simultaneous implementation of both policies would present great synergies: mutual strengthening and exponentiation of the health and financial protection benefits (eg, poverty cases averted); compensation of lost revenues by smoke–free places by increases in excise taxes; and providing a resulting combined pro–poor policy where the potentially flat distribution of benefits of smoke–free places are compensated by increases in excise taxes, and increases in taxes to the poor are compensated by reductions in cigarette spending due to bans.
