# Supplementary material for: Distributional benefits of tobacco tax and smoke–free workplaces in China: A modeling study
Source: J Glob Health. 2017 Nov 10;7(2):020701. doi: 10.7189/jogh.07.020701 (PMC5681709; doi:10.7189/jogh.07.020701)
Supplement: Online Supplementary Document [file jogh-07-020701-s001.pdf]

J Global Health 2017;7:020701

Online supplementary document - "Distributional benefits of tobacco tax and smoke-free workplaces in China: a modeling study"

1. Mathematical derivations

We adapted an existing extended cost-effectiveness analysis (ECEA) framework of tobacco taxation in China[1] and developed it further in simulating and comparing two key policies: a large increase in excise taxes, raising the share of all applicable taxes of the retail price of tobacco products to 75%; and an implementation of total smoking bans in workplaces.

The Chinese male population is divided into five-year age groups from age 0 to age 84, with an additional age group including everyone above age 85. We distinguish between the "current smokers" i.e. those individuals aged 15 and over, and the "future smokers" i.e. those individuals aged under 15. The population is also divided into income quintiles.

For each tobacco control policy examined, we estimate, independently, at the population level:

(i) the number of premature deaths averted due to smoking cessation;

(ii) the net change in tax revenues;

(iii) the financial risk protection provided to the population by preventing the out-of-pocket (OOP) medical expenditures related to treatment of tobacco-related disease, hence the associated numbers of cases of poverty averted and catastrophic health expenditures averted.

1.1. Premature deaths averted

Excise tax increase. At a given age a, the number of individuals who quit is driven by the participation elasticity (assumed to be half of price elasticity).[1-3] The price elasticity, denoted $\varepsilon_{q,a}$, varies by income quintile q and by age a. ε is assumed to be twice as large among the youth (15-

24 year-olds and the current under 15 year-olds; Table 1 in the main text).[1-4] After price increase, the number of smokers then becomes:

$$S_{post,a,q} = \left(1 + \frac{1}{2}\varepsilon_{q,a}\Delta P\right) S_{ante,a,q} \ , \qquad (1)$$

where $S_{ante,a,q}$ is the number of smokers in the age group a and income quintile q before price increase, and ΔP is the relative change in the retail price of cigarettes (here 75%, which raises the mean price per pack from \$2.00 to \$3.50; Table 1 in the main text).

Workplace total bans. The number of individuals who quit at age a is related to the relative reduction in smoking prevalence, denoted ΔR (9%; Table 1 in the main text), which we assumed not to vary by income quintile q (nor by age a). After ban implementation, the number of smokers then becomes:

$$S_{post,a,q} = (1 - \Delta R) S_{ante,a,q} \ , \qquad (2)$$

where $S_{ante,a,q}$ is the number of smokers in the age group a and income quintile q before ban.

Subsequently, for each policy, the number of premature deaths averted would be:

$$D_{a,q} = \left(S_{ante,a,q} - S_{post,a,q}\right) \delta RR(a) \ , \qquad (3)$$

where δ is the probability that a continuing smoker will die prematurely (0.50 according to Doll et al[5]) and $RR(a)$ is the relative risk reduction of premature mortality depending on age at quitting a. Based on the age-specific relative risk reductions from Doll et al,[5] we modeled a continuous $RR(a)$ (for every five-year age group) among former smokers using cubic splines, which gave, for the age groups $\{0-5; 5-10; 10-15; 15-20; 20-25; 25-30; 30-35; 35-40; 40-45; 45-50; 50-55; 55-60; 60-65; 65-70; 70-75; 75-80; 80-85; >85\}$, the vector:

$RR(a) =$

$\{1; 1; 1; 0.97; 0.95; 0.92; 0.89; 0.87; 0.84; 0.79; 0.73; 0.63; 0.50; 0.36; 0.25; 0.16; 0.09; 0.05\}$.

1.2. Net change in tax revenues

Excise tax increase. After price increase and cigarette consumption reduction, the change in tax revenues in quintile q is given by:

$$TR_q = \sum_a S_{ante,a,q} Cig[t_2(1 + \varepsilon_{q,a}\Delta P) - t_1], \quad (4)$$

where Cig is the number of cigarette packs consumed per individual per year, t_1 is the tax share per cigarette pack before tax hike (\$1.12 or 56% of \$2.00), and t_2 is the tax share per cigarette pack after tax hike (\$2.63 or 75% of \$3.50).

Workplace total bans. After smoking prevalence reduction, the change in tax revenues in quintile q is given by:

$$TR_q = \sum_a S_{ante,a,q} Cig[t_1(1 - \Delta R)], \quad (5)$$

where we recall $S_{ante,a,q}$ is the number of smokers in the age group a and income quintile q before policy, and ΔR is the relative reduction in smoking prevalence.

1.3. Financial risk protection

From the number of tobacco-related premature deaths averted by each policy (estimated using equation (3) above), we derive the share of these deaths attributable to neoplasms, stroke, ischemic heart disease, and chronic obstructive pulmonary disease (see Table 1 in the main text). Then, based on these causes of death, we assign OOP treatment-related costs, accounting for healthcare utilization and reimbursement by insurance.[6]

The OOP treatment-related costs averted in quintile q are given by:

$$OOP_q = \sum_a D_{a,q} \sum_d P_d u_{d,q} c_d (1 - f) \ , \quad (6)$$

where $D_{a,q}$ is the number of premature deaths averted in quintile q depending on age at quitting a, P_d is the share of disease d to the total tobacco-related premature deaths, c_d is the treatment cost of disease d, $u_{d,q}$ is healthcare utilization for disease d in quintile q, and f is the fraction reimbursed by insurance (48%; Table 1 in the main text).

Subsequently, we estimated the number of poverty cases attributed to OOP_q costs (equation (6) above) that would be averted by each policy. To do so, we counted the number of individuals for whom OOP direct medical costs $c_q = (1 - f)c_d$ would be incurred, which corresponded to $\sum_a D_{a,q} \sum_d P_d u_{d,q}$ individuals. Among those individuals, we counted those for which: (i) $y > P_l$,

and (ii) $y - c_q < P_l$, where P_l was the poverty line threshold and y was their income. We used a poverty line threshold P_l of US$1.90 per day (or US$694 annually).

Concerning y, as there was no income distribution readily available for China, we derived a simulated distribution of income drawn from a simulated gamma distribution[8,9] whose shape and scale parameters were based on income per capita (US$3039, the mean of the distribution) and Gini coefficient (0.43, available from China's National Bureau of Statistics and the World Bank).[7,10] For each occurrence of OOP direct medical costs, we sampled an annual income y extracted from the income distribution. Subsequently, we could estimate the number of individuals (among those $\sum_a D_{a,q} \sum_d P_d u_{d,q}$) for whom the size of OOP direct medical costs c_q would push them under the poverty line threshold P_l. A poverty case was counted when first individual income was above the poverty line ($y > P_l$) and second individual income minus OOP direct medical costs was below the poverty line ($y - c_q < P_l$).

Likewise, we estimated the number of cases of catastrophic expenditures attributed to OOP_q costs (equation (6) above) that would be averted by each policy. To do so, we counted the number of individuals for whom OOP direct medical costs $c_J = (1 - f)c_d$ would be incurred, which corresponded to $\sum_a D_{a,q} \sum_d P_d u_{d,q}$ individuals. Among those individuals, we counted those for whom OOP_q would exceed 10% of simulated annual income, i.e.: $OOP_q > 0.10 * y$.

2. **Sensitivity analyses.**

Four sensitivity analyses were conducted to test key scenarios and parameters.

First, for the excise tax increase, the model was run with a flat price elasticity across quintiles (Table S1). The results are presented and compared with the base case scenario (Table 1 in the main text) in Figure S1 below.

Table S1. Flat price elasticity of demand for tobacco products by age group and income quintile, which was assumed in the sensitivity analysis.

Age group	Average	Income quintile I	Income quintile II	Income quintile III	Income quintile IV	Income quintile V
≥ 25 year-olds	-0·38	-0·38	-0·38	-0·38	-0·38	-0·38
15-24 year-olds	-0·76	-0·76	-0·76	-0·76	-0·76	-0·76
Future smokers i.e. under 15 year-olds	-0·76	-0·76	-0·76	-0·76	-0·76	-0·76

Second, we tested the impact of brand switching by incorporating a parameter S_w, capturing the proportion of smokers (proportions of $S_w = 0.33$ or $S_w = 0.75$ were tested) who would respond to price increase by switching to a cheaper cigarette brand instead of quitting or decreasing consumption. In this situation, the number of smokers after retail price increase in age group a in income quintile q would become:

$$S_{post,a,q} = \left(1 + \frac{1}{2}\varepsilon_{q,a}\Delta P(1 - S_w)\right) S_{ante,a,q} \,, \qquad (7)$$

where we recall $S_{ante,a,q}$ is the number of smokers in age group a in income quintile J without price increase, and ΔP is the relative change in the price of cigarettes. Furthermore, in a simple way (likely overestimation), the change in tax revenues in income quintile q could be assumed as:

$$R_q = \sum_a S_{ante,a,q} Cig[t_2(1 + \varepsilon_{q,a}\Delta P(1 - S_w)) - t_1], \qquad (8)$$

where we recall Cig is the number of cigarette packs consumed per individual per year, t_1 is the tax share per cigarette pack before tax hike, and t_2 is the tax share per cigarette pack after tax hike. Here, we implicitly assume that the proportions of smokers who switch (S_w) would reduce the price elasticity of demand for tobacco products (to -0.27 and -0.10 for $S_w = 0.33$ and $S_w = 0.75$, respectively); and that the newer estimation of poverty cases averted would follow the newer formulation of premature deaths averted following (7). Further sensitivity analyses could have been pursued where switching would vary with income (i.e. $S_{w,q}$), yet the lack of empirical evidence prevented us to do so. The results are presented and compared with the base case scenario (Table 1 in the main text) in Figure 6 (main text).

Third, for workplace total bans, we tested an alternative effect size assuming an absolute reduction in prevalence of 3.8% and a decrease in average consumption of 3.1 cigarettes per day among continuing smokers using a meta-analysis based on findings from four countries.[11] In this case, the absolute reduction in prevalence was further adjusted to 2.2% accounting for the number of workplaces already having full smoking bans (i.e. 31%)[12] and for the number of men under age 60 employed (i.e. 82%).[13] The results are presented and compared with the base case scenario in Figure S2. Insufficient evidence however prevented us from testing the impact of a differential

effect size per income quintile q. Hence, we pursued a sensitivity analysis where we decreased by 50% the relative smoking prevalence reduction of 9.0% (base case scenario) in the bottom income quintile, accounting for the possibility that smokers in the bottom income quintile may not be employed in the formal sector where such smoking bans could be enacted. The results are presented and compared with the base case scenario in Figure S2.

Fourth, for both excise tax increase and workplace smoking bans, we tested two alternative poverty thresholds of $= US\$1$ and $= US\$3$ per day, respectively. The results are presented and compared with the base case scenario in Figure S3 below.

Figure S1. Impact of a 75% increase in the retail price of cigarettes through excise tax (price elasticity of demand for cigarettes varying by income quintile, or either flat price elasticity) in China, per income quintile, on: the number of tobacco-related premature deaths averted (a); the net change in tax revenues collected on cigarette sales on current smokers (15 years of age and above) (b); the amount of out-of-pocket expenditures related to tobacco-related disease treatment costs averted (c); the number of tobacco-related poverty cases averted due to the prevention of out-of-pocket tobacco-related disease treatment costs (d); and the number of averted cases of catastrophic expenditures due to the prevention of out-of-pocket tobacco-related disease treatment costs (e).

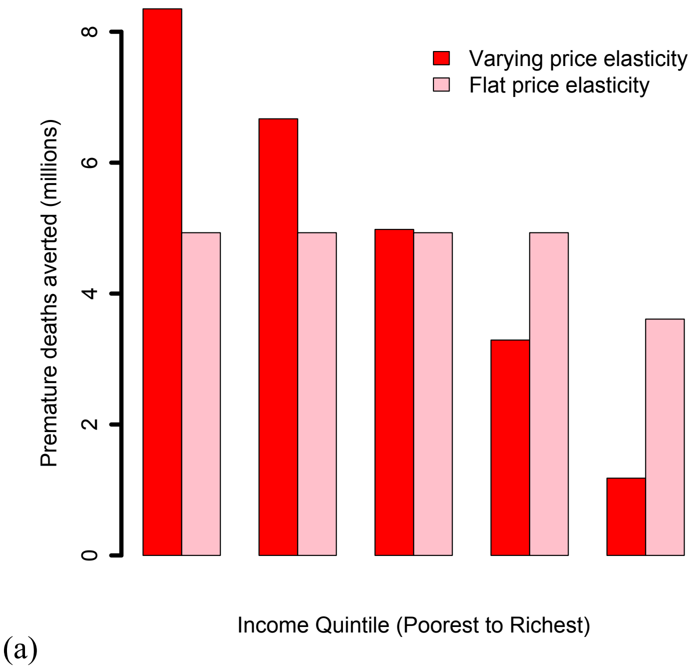

(a)

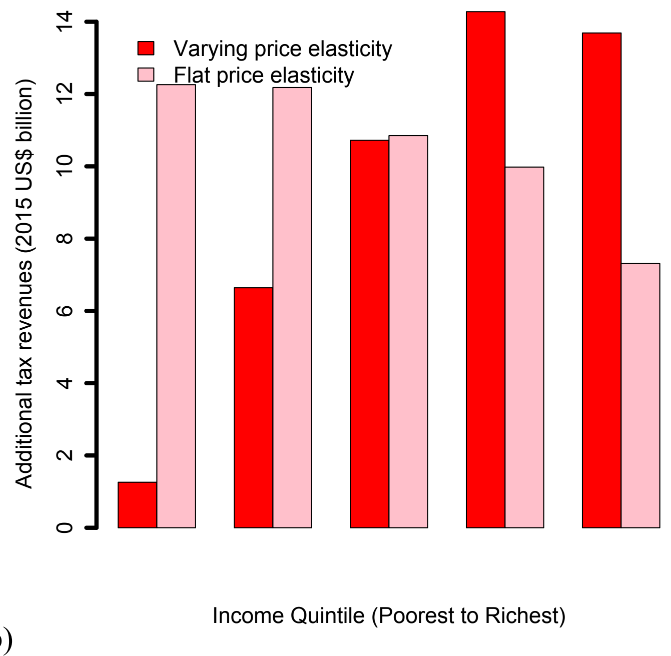

(b)

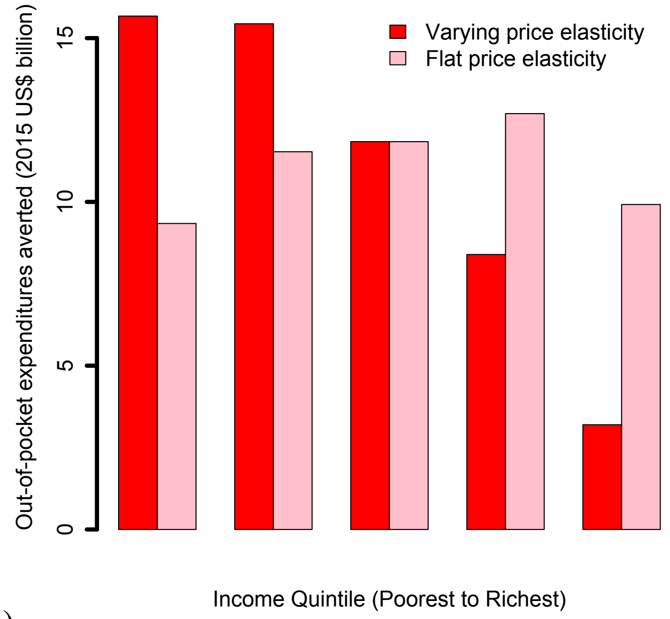
Out-of-pocket expenditures averted (2015 US$ billion)
0
5
10
15
Varying price elasticity
Flat price elasticity
Income Quintile (Poorest to Richest)

(c)

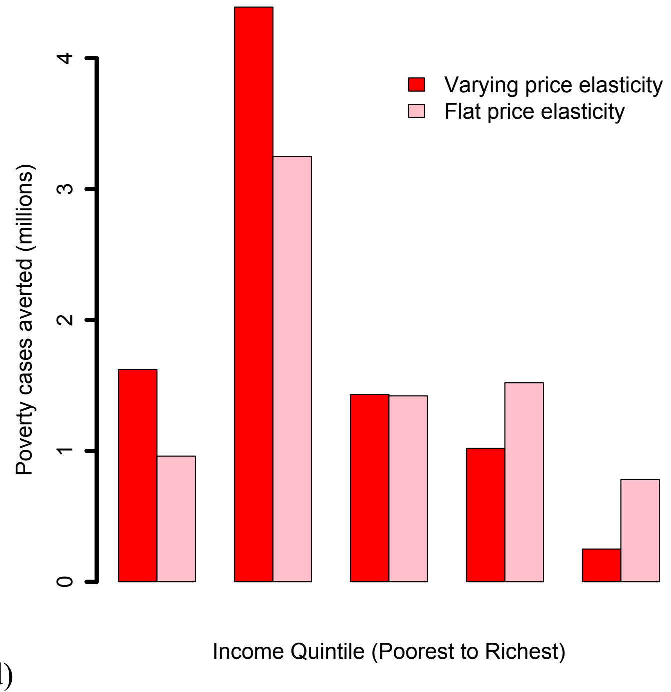
Poverty cases averted (millions)
0
1
2
3
4
Varying price elasticity
Flat price elasticity
Income Quintile (Poorest to Richest)

(d)

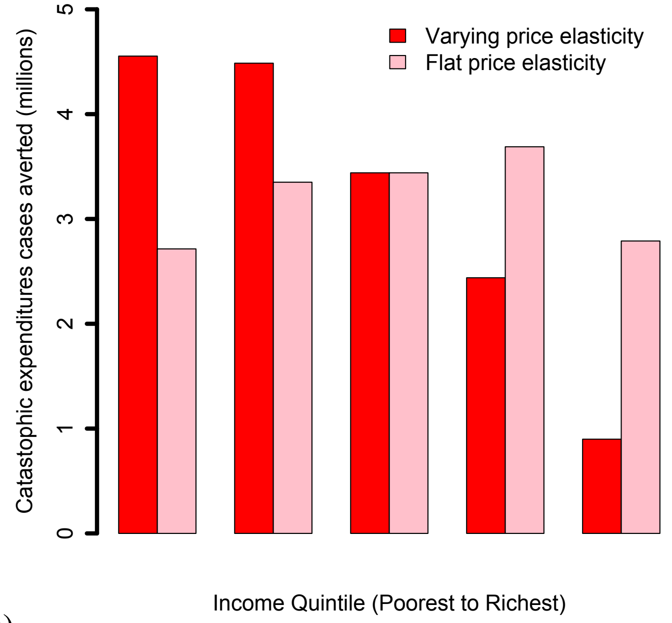
Catastophic expenditures cases averted (millions)
0
1
2
3
4
5
Varying price elasticity
Flat price elasticity
Income Quintile (Poorest to Richest)

(e)

Figure S2. Impact of workplace total smoking bans in China, per income quintile, on: the number of tobacco-related premature deaths averted (a); the net change in tax revenues collected on cigarette sales on current smokers (15 years of age and above) (b); the amount of out-of-pocket tobacco-related disease treatment costs averted (c); the number of tobacco-related poverty cases averted due to the prevention of out-of-pocket tobacco-related disease treatment costs (d); and the number of cases of catastrophic expenditures averted due to the prevention of out-of-pocket tobacco-related disease treatment costs (e). Three distinct effect sizes were tried: (1) relative smoking prevalence reduction of 9.0% (base case scenario); (2) absolute smoking prevalence reduction of 2.2% and absolute consumption reduction by 3.1 cigarettes per day (sensitivity analysis); and (3) relative smoking prevalence reduction of 4.5% in the bottom income quintile as opposed to 9.0% in all the other income quintiles.

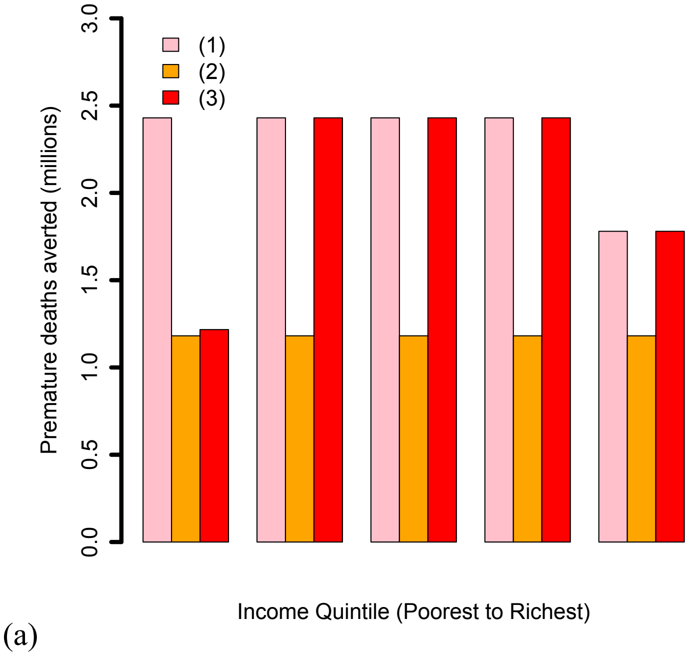

(a)

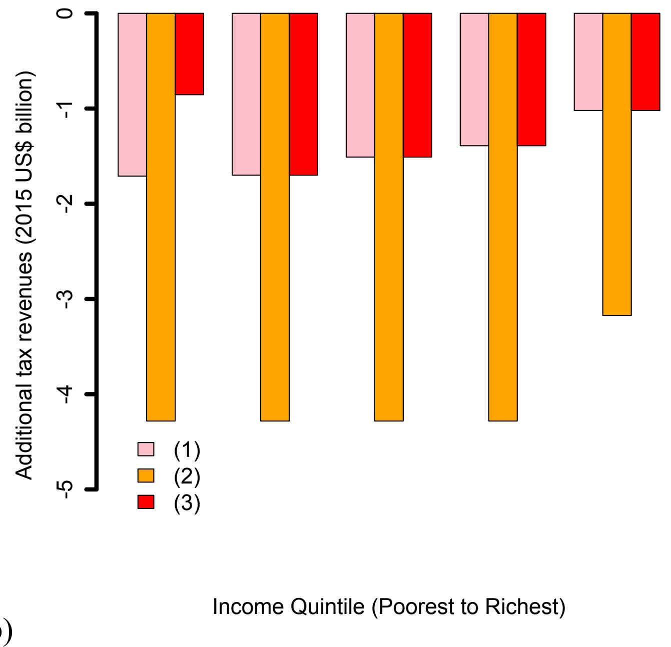

(b)

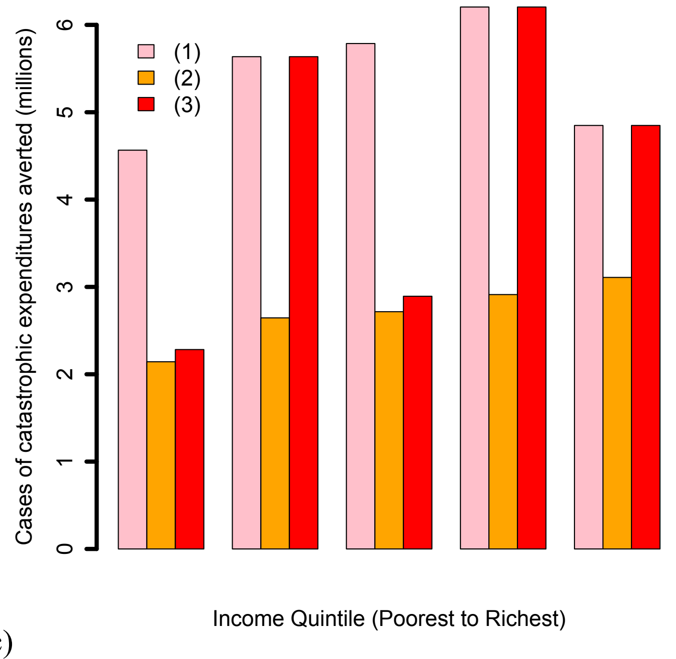
Cases of catastrophic expenditures averted (millions)
0
1
2
3
4
5
6
(1)
(2)
(3)
Income Quintile (Poorest to Richest)
(c)

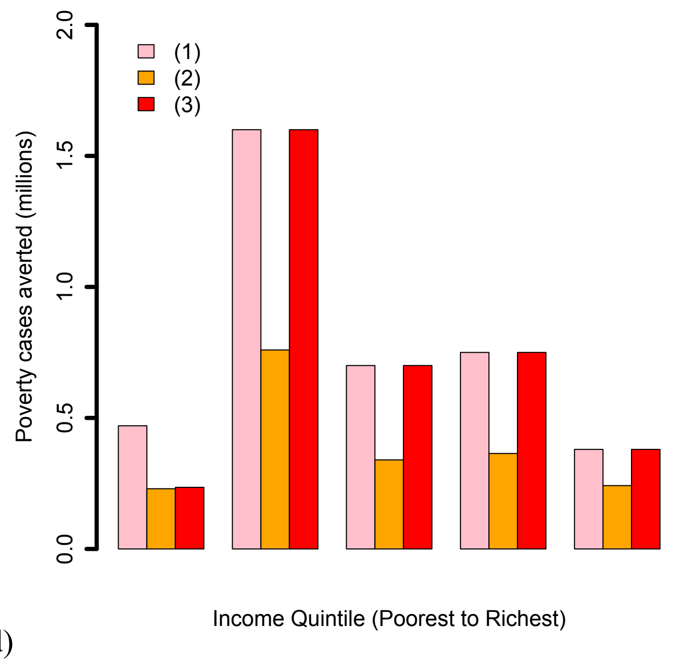
Poverty cases averted (millions)
0.0
0.5
1.0
1.5
2.0
(1)
(2)
(3)
Income Quintile (Poorest to Richest)
(d)

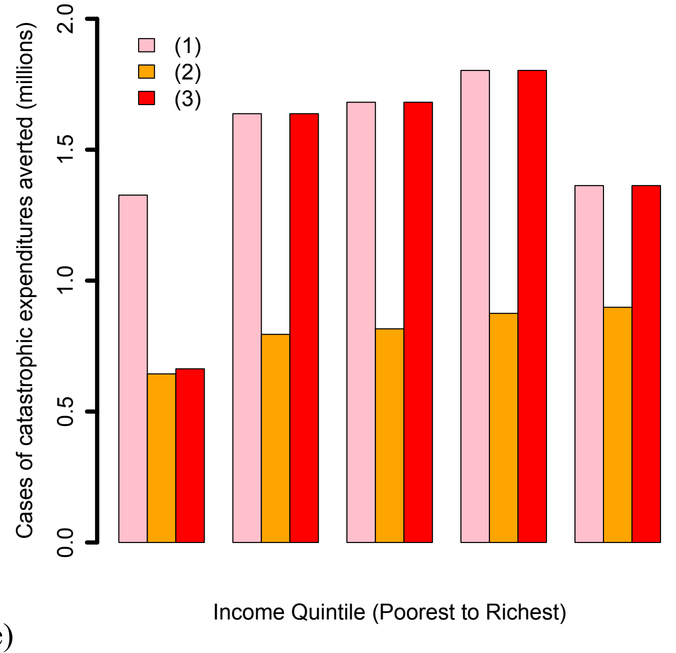
Cases of catastrophic expenditures averted (millions)
0.0
0.5
1.0
1.5
2.0
(1)
(2)
(3)
Income Quintile (Poorest to Richest)
(e)

Figure S3. Impact of tobacco control policies (75% increase in the retail price of cigarettes through excise tax (a); workplace smoking total bans (b)) in China, per income quintile, on the number of tobacco-related poverty cases averted due to the prevention of out-of-pocket tobacco-related disease treatment costs, using three distinct poverty thresholds: US$1, US$1.90, and US$3 per day.

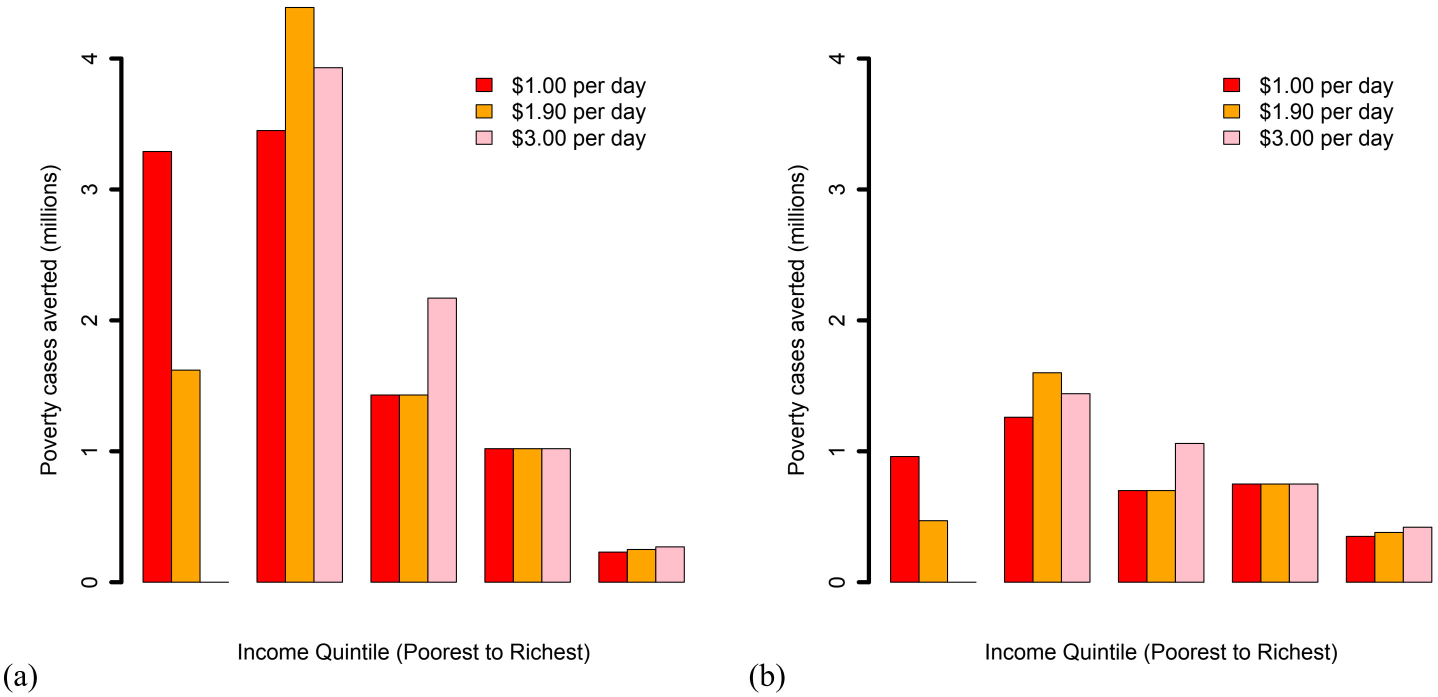

(a) (b)

3. References

1. Verguet S, Gauvreau CL, Mishra S, et al. The consequences of tobacco tax on household health and finances in rich and poor smokers in China: an extended cost effectiveness analysis. Lancet Global Health 2015; 3(4):e206-16.
2. Jha P, Joseph R, Liu D, et al. Tobacco taxes: a win-win measure for fiscal space and health. Mandaluyong City, Philippines: Asian Development Bank, 2012.
3. International Agency for Research on Cancer, World Health Organization. IARC handbooks of cancer prevention, tobacco control, volume 14 – Effectiveness of tax and price policies for tobacco control. Lyon: World Health Organization, 2011.
4. World Health Organization. WHO technical manual on tobacco tax administration. Geneva: World Health Organization, 2010.
5. Doll R, Peto R, Boreham J, Sutherland I. Mortality in relation to smoking: 50 years' observations on male british doctors. British Medical Journal 2004; 328(7455):1519.
6. Yip WC, Hsiao WC, Chen W, et al. Early appraisal of china's huge and complex health-care reforms. Lancet 2012; 379(9818):833-842.
7. World Bank. World development indicators. http://data.worldbank.org/data-catalog/world-development-indicators (accessed December 9, 2013).
8. Salem, ABZ and Mount, TD. A convenient descriptive model of income distribution: the gamma density. Econometrica 1974; 42:1115–1127.
9. Kemp-Benedict E. Income distribution and poverty – Methods for using available data in global analysis. May 17, 2001. Available from: http://gdrs.sourceforge.net/docs/PoleStar_TechNote_4.pdf (accessed November 26, 2016).
10. National Bureau of Statistics of China. China Statistical Yearbook 2014, Table 6-1 per capital income and consumption expenditure nationwide. Available from: http://www.stats.gov.cn/tjsj/ndsj/2014/zk/html/Z0601e.htm (accessed November 26, 2016).
11. Fichtenberg CM, Glantz SA. Effect of smoke-free workplaces on smoking behaviour: systematic review. British Medical Journal 2002; 325 (7357):188.
12. Global Adult Tobacco Survey. Global Adult Tobacco Survey China 2010 Country Report. Geneva: World Health Organization, 2010.
13. China Labour Bulletin. 2014. Employment in China, 22 June, 2013 2013. Available from http://www.clb.org.hk/en/content/employment-china. [cited Jan 15 2014].
